# Radiation Therapy for Grade 3 Gliomas: Correlation of MRI Findings With Prognosis

**DOI:** 10.7759/cureus.16887

**Published:** 2021-08-04

**Authors:** Masashi Mizumoto, Hsiang-Kuang Liang, Yoshiko Oshiro, Masahide Matsuda, Hidehiro Kohzuki, Takashi Iizumi, Haruko Numajiri, Kei Nakai, Toshiyuki Okumura, Eiichi Ishikawa, Hideyuki Sakurai

**Affiliations:** 1 Radiation Oncology, University of Tsukuba Hospital, Tsukuba, JPN; 2 Department of Biomedical Engineering, National Taiwan University, Taipei, TWN; 3 Division of Radiation Oncology, National Taiwan University Hospital, National Taiwan University College of Medicine, Taipei, TWN; 4 Radiation Science and Proton Therapy Center, National Taiwan University College of Medicine, Taipei, TWN; 5 Department of Radiation Oncology, Tsukuba Medical Center Hospital, Tsukuba, JPN; 6 Neurosurgery, University of Tsukuba Hospital, Tsukuba, JPN; 7 Department of Radiation Oncology, Proton Medical Research Center, University of Tsukuba Hospital, Tsukuba, JPN; 8 Department of Radiation Oncology, University of Tsukuba Hospital, Tsukuba, JPN; 9 Radiation Oncology, Tsukuba University, Tsukuba, JPN

**Keywords:** glioma, high grade glioma, grade 3, radiotherapy, risk factors

## Abstract

Background and objective

Postoperative radiotherapy is usually indicated for both grade 3 glioma and grade 4 glioblastoma. However, the treatment results and tumor features of grade 3 glioma clearly differ from those of glioblastoma. There is limited information on outcomes and tumor progression for grade 3 glioma. In this study, we evaluate the result of postoperative radiotherapy for grade 3 glioma and focus on the correlation of MRI findings with prognosis.

Methods

In this study, 99 of 110 patients with grade 3 glioma who received postoperative radiotherapy and were followed up for more than one year were retrospectively analyzed. The total irradiation dose was 60.0 Gy in 30 fractions, and daily temozolomide or two cycles of nimustine (ACNU) was concurrently administered during radiotherapy. The median follow-up period was 46 months (range: 2-151 months).

Results

In multivariate analysis, pathology [anaplastic oligodendroglioma (AO) vs. anaplastic astrocytoma (AA)], the status of surgical resection (biopsy vs. partial resection or more), and contrast enhancement (enhanced by MRI image or not) were significant factors for overall survival (OS). The five-year OS for AO vs. AA cases were 76.8% vs. 46.1%, total to partial resection vs. biopsy cases were 72.7% vs. 21.0%, and non-enhanced vs. enhanced cases were 82.5% vs. 45.6%, respectively. In multivariate analysis, the status of surgical resection and longer extension of preoperative edema (PE) were significant factors for progression-free survival (PFS). The five-year PFS for the total to partial resection vs. biopsy cases were 52.9% vs. 10.7%, and non-extensive PE vs. extensive PE (EPE) cases were 62.2% vs. 19.1%, respectively.

Conclusion

Our results suggest that a contrast-enhanced tumor on MRI and a longer PE may also be significantly associated with OS and PFS among grade 3 glioma patients.

## Introduction

Grade 3 glioma, including anaplastic astrocytoma (AA) and anaplastic oligodendroglioma (AO), is classified as a high-grade glioma. Postoperative radiotherapy is usually indicated for both grade 3 glioma and grade 4 glioblastoma. However, the treatment results and tumor features of grade 3 glioma clearly differ from those of glioblastoma, but there is scarce data on outcomes and tumor progression for grade 3 glioma [1-7].

In imaging, astrocytoma does not show enhancement, and oligodendroglioma shows minimal to moderate patchy multifocal enhancement in up to 50% of tumors. White et al. have suggested that contrast enhancement is a typical feature of high-grade tumors for AO [1]. Recently, Liang et al. suggested that longer extension of preoperative edema (PE) was significantly correlated with the prognosis of glioblastoma [2]. Grade 3 glioma sometimes also shows long PE, but its significance is unclear. We have conducted three-dimensional conformal radiotherapy (3D-CRT) for grade 3 glioma with concurrent chemotherapy postoperatively. In this study, we report the outcomes of grade 3 gliomas treated with radiotherapy, and we focus on identifying factors correlated with the treatment results, including longer PE and enhancement regions.

## Materials and methods

Patients

Between 2005 and 2016, 110 patients with grade 3 glioma received postoperative radiotherapy at our center. In this study, 99 of these patients who were followed up for more than one year were retrospectively analyzed (patients who died within one year were included). The patient characteristics are shown in Table 1.

**Table 1:** Patient characteristics KPS: Karnofsky performance status; PE: preoperative edema

Characteristics	Values
Age in years, <60, ≥60	21-82 (median: 55) 55, 44
Gender, n	
Male	54
Female	45
KPS, n	
100	30
90	22
80	20
40-70	27
Surgical status, n	
Gross total resection	11
Subtotal resection	24
Partial resection	39
Biopsy	25
Pathology, n	
Anaplastic astrocytoma	56
Anaplastic oligodendroglioma	43
Gd-enhanced, n	
No	36
Yes	63
PE, n	
<2.0 cm	53
≥2.0 cm	46

**Figure 1:**
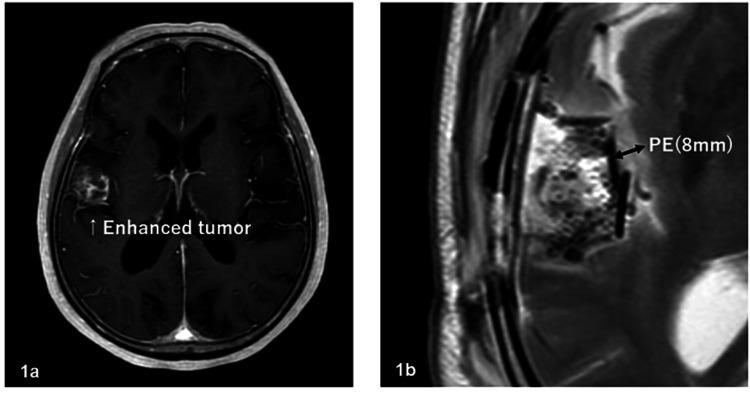
Supplementary MRI images – 1 1a: Gd-enhanced tumor before surgery; 1b: 8-mm PE after surgery MRI: magnetic resonance imaging; PE: preoperative edema

**Figure 2:**
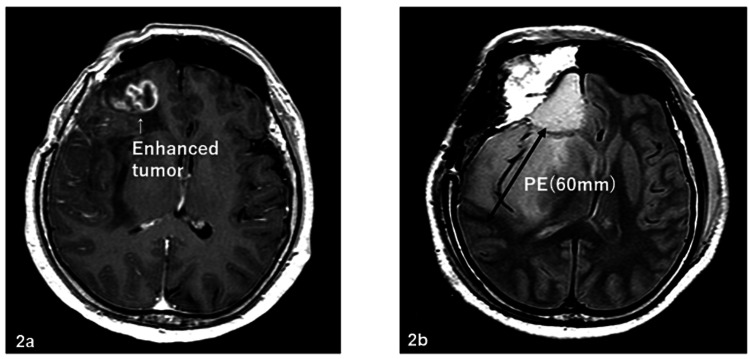
Supplementary MRI images – 2 2a: Gd-enhanced tumor before surgery; 2b: 60-mm PE after surgery MRI: magnetic resonance imaging; PE: preoperative edema

**Figure 3:**
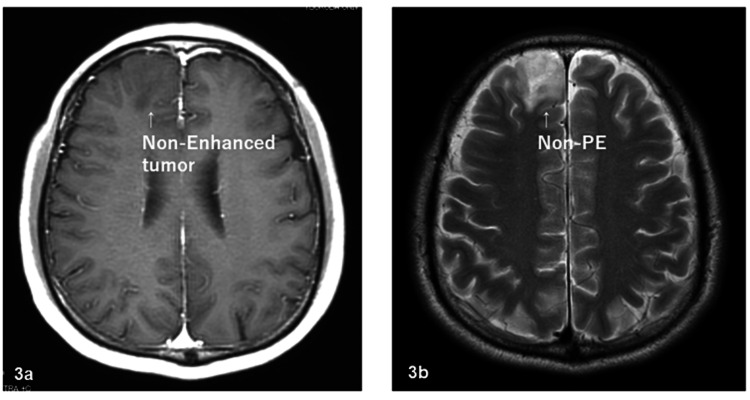
Supplementary MRI images – 3 3a: non-enhanced tumor before surgery; 3b: non-PE before surgery. The tumor shapes of 3a and 3b were almost the same MRI: magnetic resonance imaging; PE: preoperative edema

**Figure 4:**
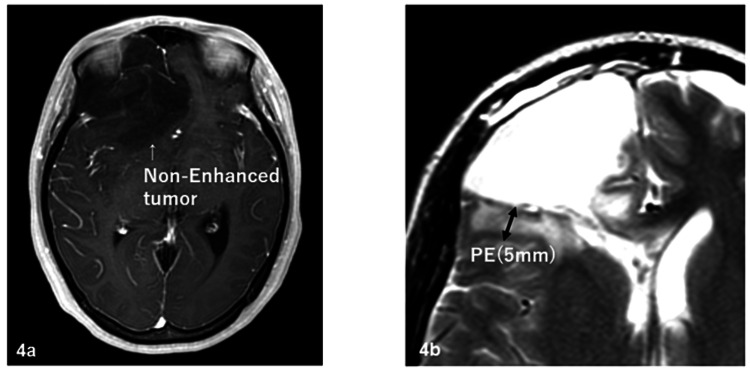
Supplementary MRI images – 4 4a: non-enhanced tumor before surgery; 4b: 5-mm PE after surgery MRI: magnetic resonance imaging; PE: preoperative edema

The patients included 54 males and 45 females, and the median age at the start of radiotherapy ranged from 21 to 82 years (median: 55 years). The Karnofsky performance status (KPS) was 40-70, 80, 90, and 100 for 27, 20, 22, and 30 patients, respectively. The surgical status was biopsy, partial resection, subtotal resection (STR), and total resection for 25, 39, 24, and 11 patients, respectively; and the pathological results, reviewed by two or more physicians, were AO for 43 patients and AA for 56 patients. On MRI with contrast enhancement before surgery, tumors were classified into enhanced (n=63) and non-enhanced (n=36) groups. On MRI before surgery, PE regions were defined as a hyperintense area on T2-weighted or fluid-attenuated inversion recovery (FLAIR) MRI or a hypointense area on CT. PE was observed in 72 patients and was absent in 27 patients. The maximum range of PE was 0-9.87 cm and the mean range was 1.98 cm in all patients. Extensive PE (EPE) was defined as PE extending at least 2 cm from the tumor edge. Figures 1-4 show the supplementary MRI images.

Treatment

All patients received radiotherapy after surgery. Surgical status was classified as gross total resection (GTR), STR, partial resection, and biopsy according to neurosurgeon records. Enhanced MRI was performed within three days after surgery. Treatment planning involved the use of CT images at 3-mm intervals at the treatment position. Based on preoperative MRI, the clinical target volume (CTV)-1 included the entire surgical cavity and surrounding edema plus a 1.2-cm margin. The CTV-2 included the residual tumor or cavity plus a 1.2-cm margin. In the first 23-25 fractions, radiotherapy of 2.0 Gy per fraction was delivered to CTV-1, and in the last five to seven fractions, the CTV was shrunk to CTV-2. The total irradiation dose was up to 60.0 Gy in 30 fractions. The planning target volume (PTV) was defined as the CTV plus 3 mm for setup error. The exposure dose limit was adjusted to ≤10 Gy for the lens, ≤44 for the retina, ≤50 Gy for the chiasm and optic nerve, and ≤60 Gy for 1/5 of the brainstem [3,4]. If the dose to an organ at risk exceeded the exposure dose limit, the margin of the lesion was reduced. Chemotherapy with daily temozolomide (75 mg/m^2^) for 28 days or two cycles of nimustine (ACNU) (80 mg/m^2^) was concurrently administered during radiotherapy [3-5].

Statistical analysis

Data analysis and statistical tests were performed using SPSS Statistics version 27 (IBM, Armonk, NY). Overall survival (OS) was calculated from the date of surgery to death. Progression-free survival (PFS) was calculated from the date of surgery to disease progression, including death or tumor progression proven by imaging. Survival was calculated using the Kaplan-Meier method. Differences in survival were compared between the groups by log-rank test. Univariate and Cox regression analyses were performed to evaluate factors that influenced survival, including age (<60 years vs. ≥60 years), gender, KPS (≤70 vs. ≥80), enhanced region (yes vs. no), status of tumor resection (biopsy vs. partial resection), and PE (<2 cm vs. ≥2 cm). Genetic information was not evaluated because only 26 patients had O6-methylguanine-DNA methyltransferase promoter (MGMT) information and six had 1p/19q codeletion information, respectively.

Ethical approval

The Ethics and Steering Committees of Tsukuba approved this study and written informed consent was obtained from every patient prior to radiotherapy. The study was conducted in accordance with the Declaration of Helsinki, and the protocol was approved by the Institutional Review Board (Tsukuba Clinical Research & Development Organization, H30-114).

## Results

**Figure 5:**
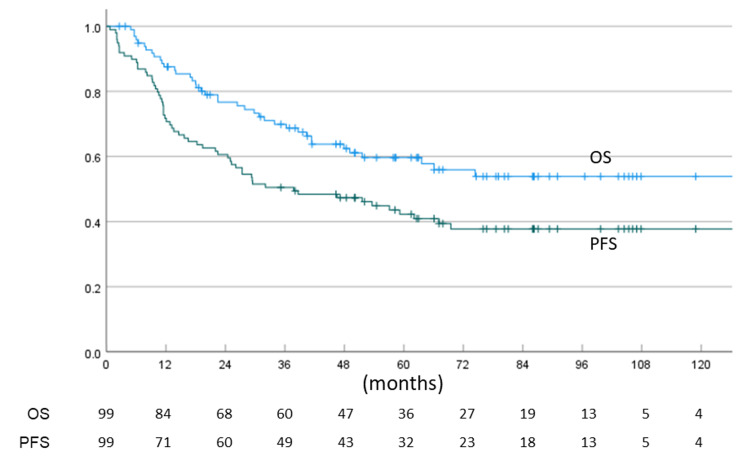
The overall survival (OS) and progression-free survival (PFS) for all patients

The median follow-up period was 46.3 months (range: 2.6-151.9 months). Sixty patients were alive at the last follow-up. The one-, three-, five-, and seven-year OS for all patients was 87.6% (95% CI: 81.0-94.1), 69.9% (60.5-79.3), 59.7% (49.3-70.0), and 53.9% (42.6-65.1), respectively, with a mean OS time of 95.3 months (81.8-108.8). The one-, three-, five-, and seven-year PFS for all patients was 71.7% (62.8-80.6), 50.5% (40.7-60.3), 42.3% (32.3-52.2), and 37.8% (27.6-47.9), respectively, with a mean PFS time of 66.8 months (54.1-79.5). The OS and PFS for all patients are shown in Figure 5.

In univariate analysis, the significant factors for OS were age (p=0.031), status of surgical resection (biopsy vs. partial resection, STR, and GTR, p=0.001), pathology (AO vs. AA, p=0.005), KPS (40-70 vs. 80-100, p=0.001), enhancement (p=0.001), and EPE (≤2 cm vs. >2 cm, p=0.001). In multivariate analysis, pathology (p=0.029), status of surgical resection (p=0.005), and contrast enhancement (p=0.045) were significant factors (Table 2).

**Table 2:** Multivariate analysis of potential predictive factors for overall survival AO: anaplastic oligodendroglioma; AA: anaplastic astrocytoma; KPS: Karnofsky performance status; MST: median survival time; PE: preoperative edema

Variable	No. of patients	MST (months)	Three-year survival (%)	Five-year survival (%)	Standard error	P-value	Hazard ratio	95% CI
									Age in years								
<60	55	-	75.7	69.4	0.384	0.909	0.957	0.451-2.031
>60	44	47.9	62.1	44.6				
Gender								
Male	54	63.6	63.7	53.1	0.365	0.248	0.656	0.320-1.342
Female	45	-	76.9	66.7				
KPS								
70-100	52	-	83.9	74.5	0.409	0.478	1.337	0.600-2.980
40-60	47	38.7	53.5	41.8				
Pathology								
AO	43	-	85.3	76.8	0.386	0.029	2.315	1.087-4.928
AA	56	41.4	57.8	46.1				
Surgery								
Total-partial	74	-	81.2	72.7	0.398	0.005	3.032	1.390-6.615
Biopsy only	25	22.5	36.7	21				
PE, cm								
0-1.9	53	-	80.6	70.2	0.386	0.435	1.352	0.634-2.883
>2	46	41.4	56.6	45.7				
Gd enhancement								
No	36	-	88.6	82.5	0.462	0.045	2.53	1.022-6.262
Yes	63	49.1	58.5	45.6				

**Figure 6:**
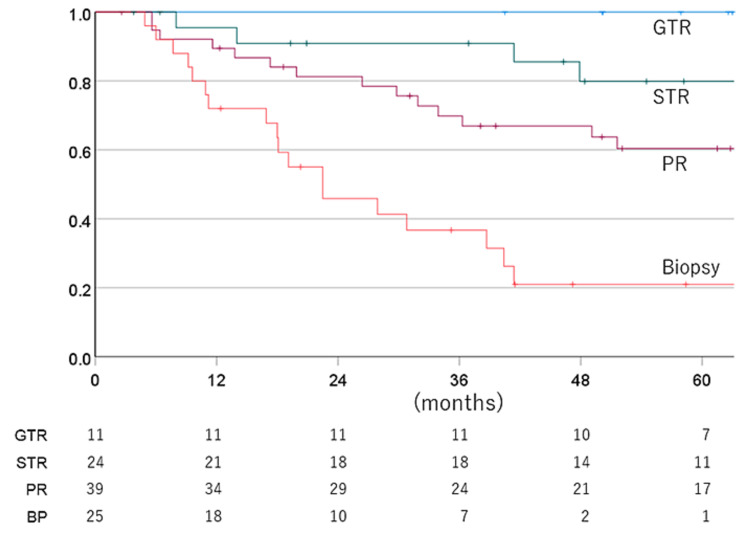
Overall survival rate for each surgical status GTR: gross total resection; STR: subtotal resection; PR: partial resection; BP: biopsy

The one-, three-, five-, and seven-year OS for AO vs. AA cases were 95.3% vs. 81.5% (one year), 85.3% vs. 57.8% (three years), 76.8% vs. 46.1% (five years), and 72.6% vs. 38.9% (seven years), with a mean OS time of 119.2 vs. 73.3 months. The one-, three-, five-, and seven-year OS for total to partial resection vs. biopsy cases were 93.0% vs. 72.0% (one year), 81.2% vs. 36.7 (three years), 72.7% vs. 21.0% (five years), and 65.4% vs. 21.0% (seven years), with a mean OS time of 111.5 vs. 33.0 months. Figure 6 illustrates OS rates for each surgical status.

The one-, three-, five-, and seven-year OS for non-enhanced vs. enhanced cases were 100% vs. 80.1% (one year), 88.6% vs. 58.5 (three years), 82.5% vs. 45.6% (five years), 73.0% vs. 42.3% (seven years), with a mean OS time of 123.1 vs. 75.2 months.

In univariate analysis, the significant factors for PFS were age (p=0.001), status of surgical resection (p=0.001), pathology (p=0.005), KPS (p=0.001), enhancement (p=0.001), and extensive PE (p=0.001). In multivariate analysis, the status of surgical resection and extensive PE were significant factors for PFS (Table 3).

**Table 3:** Multivariate analysis of potential predictive factors for progression-free survival AO: anaplastic oligodendroglioma; AA: anaplastic astrocytoma; KPS: Karnofsky performance status; MST: median survival time; PE: preoperative edema

Variable	No. of patients	MST (months)	Three-year PFS (%)	Five-year PFS (%)	Standard error	P-value	Hazard ratio	95% CI
									Age in years								
<60	55	128.2	65.5	57.4	0.308	0.331	1.35	0.738-2.470
>60	44	15.7	31.8	23				
Gender								
Male	54	27.4	46.3	39.7	0.285	0.851	0.948	0.543-1.656
Female	45	57.1	55.6	45.7				
KPS								
70-100	52	130.1	63.5	59.1	0.329	0.813	1.081	0.567-2.061
40-60	47	16.5	36.2	24.3				
Pathology								
AO	43	130.1	62.8	55.1	0.296	0.14	1.549	0.866-2.768
AA	56	25.2	41.1	32.3				
Surgery								
Total-partial	74	67.1	62.2	52.9	0.339	0.01	2.392	1.231-4.648
Biopsy only	25	11.4	16	10.7				
PE								
0-1.9 cm	53	-	64.2	62.2	0.335	0.043	1.973	1.023-3.805
≥2.0 cm	46	15.7	34.8	19.1				
Gd enhancement								
No	36	-	72.2	66.6	0.372	0.289	1.483	0.715-3.072
Yes	63	16.5	38.1	28.9				

**Figure 7:**
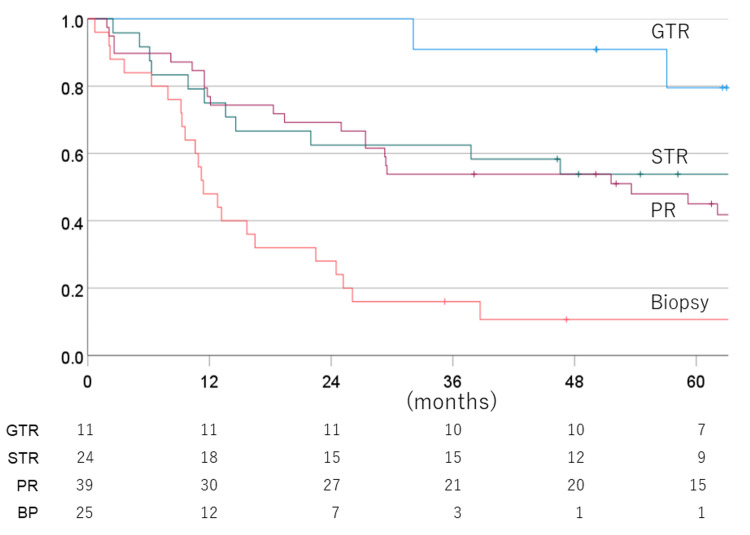
Progression-free survival rate for each surgical status GTR: gross total resection; STR: subtotal resection; PR: partial resection; BP: biopsy

The one-, three-, five-, and seven-year PFS for the total to partial resection vs. biopsy cases were 79.7% vs. 48.0% (one year), 62.2% vs. 16.0 (three years), 52.9% vs. 10.7% (five years), and 47.0% vs. 10.7% (seven years), with a mean PFS time of 80.2 vs. 20.5 months. Figure 7 depicts the PFS rate for each surgical status.

**Figure 8:**
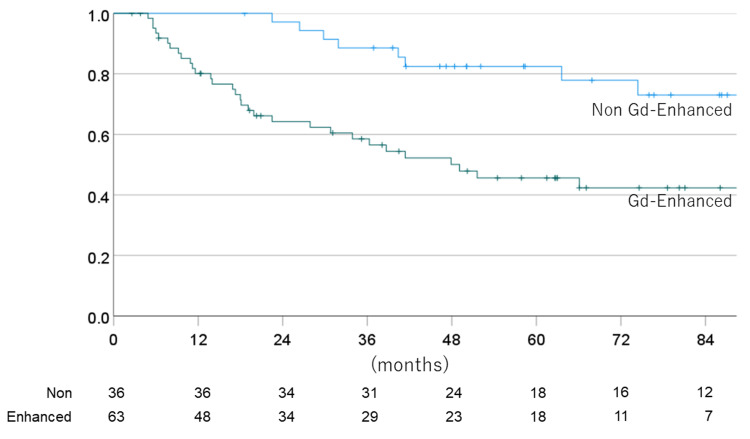
Overall survival rate for each MRI status (Gd-enhanced or non-enhanced) MRI: magnetic resonance imaging

**Figure 9:**
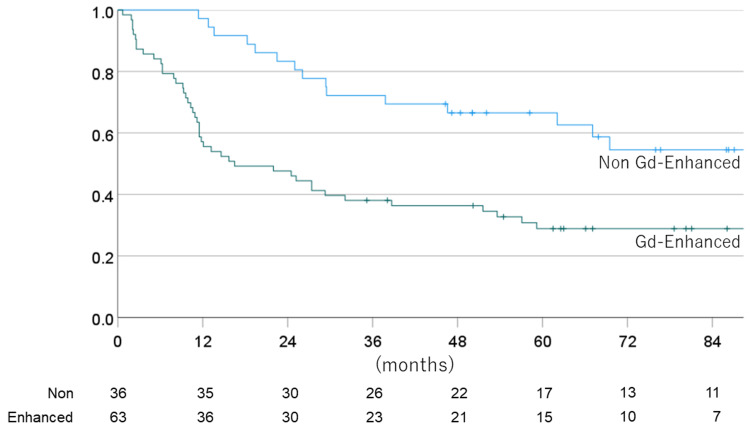
Progression-free survival rate for each MRI status (Gd-enhanced or non-enhanced) MRI: magnetic resonance imaging

**Figure 10:**
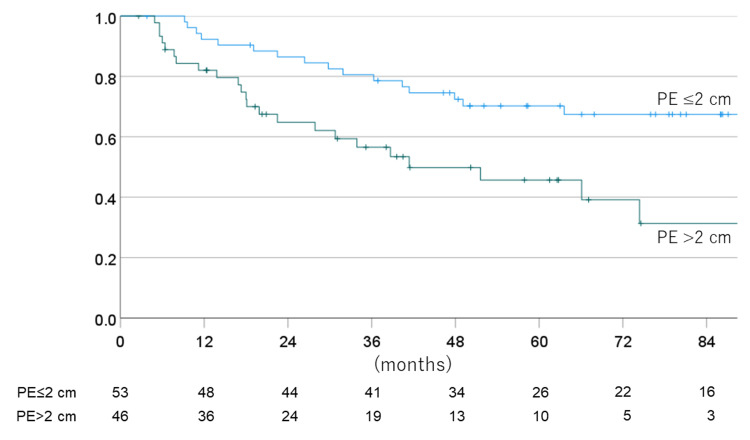
Overall survival rate for each MRI status (PE ≤2 cm or >2 cm) MRI: magnetic resonance imaging; PE: preoperative edema

**Figure 11:**
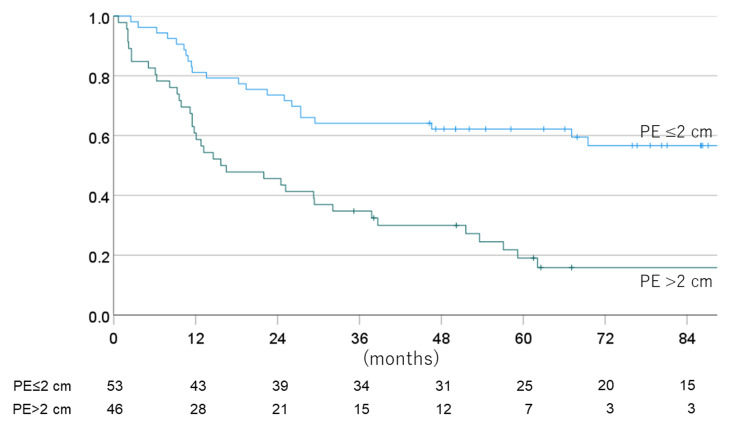
Progression-free survival rate for each MRI status (PE ≤2 cm or >2 cm) MRI: magnetic resonance imaging; PE: preoperative edema

The one-, three-, five-, and seven-year PFS for non-EPE vs. EPE cases were 81.1% vs. 60.9% (one year), 64.2% vs. 34.8 (three years), 62.2% vs. 19.1% (five years), and 56.7% vs. 15.9% (seven years), with a mean PFS time of 96.0 vs. 38.2 months. Figures 8-11 show the OS rate and PFS rate focusing on MRI image status.

## Discussion

Anaplastic gliomas account for 15-20% of all malignant gliomas. These tumors used to be included in trials together with glioblastoma as high-grade gliomas, and there have been few studies focused only on grade 3 glioma. Postoperative radiotherapy in combination with chemotherapy has improved the prognosis of high-grade glioma, with the median OS reported as two years for AA and four years for AO [6], although the prognosis is highly variable depending on factors such as the extent of resection and molecular markers [7-9]. The prognosis in our study was comparable to that in previous studies. 

In glioblastoma, it is thought that malignant cells migrate along the PE; however, the PTV margin is limited to an enhanced region plus 2 cm after 50 Gy. That is, some part of the extended edema does not receive a full dose of irradiation. Thus, Liang et al. suggested that EPE was a significant factor for the prognosis of glioblastoma and concluded that it is better to irradiate PE up to 60 Gy as much as possible [2]. In grade 3 cases, the European Organisation for Research and Treatment of Cancer (EORTC) and Radiation Therapy Oncology Group (RTOG) trial 26053 suggested that the gross tumor volume (GTV) should be defined as the entire region of high signal intensity on T2-weighted MRI or FLAIR, plus the enhanced region on preoperative CT/MRI if available, or as the enhanced region on postoperative CT/MRI if preoperative imaging is not available, plus the tumor resection margin [10]. The CTV is then defined as a 1.5-2.0-cm volumetric expansion of the GTV; the PTV adds 0.5-0.7 cm, and a total dose of 59.4 Gy is given in 33 fractions.

Minniti et al. have suggested that larger irradiated volumes could be directly responsible for clinically relevant toxicities, and isotropic expansion of 15 mm from the GTV to the CTV is now generally accepted [11]. Before these recommendations, we had used the cone down technique for grade 3 gliomas, as well as for glioblastoma [3-5]. For the initial 46-50 Gy, we defined CTV-1 as the tumor bed or cavity and surrounding edema plus 1.5-cm margins, with CTV-2 then defined as only the tumor bed or cavity plus a 1.5-cm margin for the subsequent 10-14 Gy. Therefore, when PE was extended to >2 cm, some part of the PE was excluded from CTV-2. However, our results suggested that EPE was a significant factor for a poor prognosis for PFS, but not for OS, in grade 3 glioma. In a case with EPE (>2 cm), all PE was irradiated up to 50 Gy, but part of the PE area was not irradiated after 50 Gy in this study. This may suggest that a tumor component was contained in the PE area and that 50 Gy is insufficient to control this component. The reason why OS did not decrease despite the higher recurrence rate is unclear. There may be more options for treatment after recurrence, compared to glioblastoma, and in clinical practice, we actively use retreatment methods such as surgery, reirradiation, and bevacizumab for a recurrent tumor.

To our knowledge, this is the first study of PE in grade 3 glioma, and more information is needed to determine whether the PE area should be irradiated up to 60 Gy. However, our results indicate that this approach may be better, to the extent that it is possible. Contrast enhancement reflects areas of neoangiogenesis and occurs where the blood-brain barrier is disturbed in tumor areas. However, there are few reports on the correlation between contrast enhancement and prognosis in one grade [12-16]. Lote et al. conducted a large study investigating the significance of contrast enhancement within intracranial glioma, including 947 adult patients with glioma (low grade in 336, grade 3 in 104, and glioblastoma in 481). The findings suggested that tumor contrast enhancement was strongly associated with high-grade histology, but was an independent negative prognostic factor in multivariate analysis, despite numerous previous studies finding that enhancement is associated with high-grade glioma. Among grade 3 cases, 62% showed contrast enhancement, and these patients had significantly shorter survival than those with non-enhanced tumors. Our study confirms the finding that a contrast-enhanced tumor has a poor prognosis. Thus, it may be assumed that the pathological condition of a non-enhanced tumor is closer to that of a low-grade tumor.

This study has many limitations, including its retrospective design, which prevented the evaluation of molecular factors. Of particular note, the significance of 1p/19q codeletion [17-19] and mutation of isocitrate dehydrogenase 1 or 2 (IDH1-mt or IDH2-mt) [17,19-21] has recently been established with regard to the prediction of the prognosis of gliomas. Some recent reports indicate that 1p/19q codeletion and MGMT are significantly associated with OS and PFS even in an analysis targeting only grade 3 gliomas [22-24].

## Conclusions

Based on our findings, a preoperative contrast-enhanced tumor on MRI and a longer PE may also be significantly associated with OS or PFS of grade 3 glioma. However, there is no information on genetic aspects that are considered to significantly affect the OS and PFS of grade 3 glioma. Hence, further studies are required to gain insight into the topic, especially genetic information.
